# The Potential of PD-1 and PD-L1 as Prognostic and Predictive Biomarkers in Colorectal Adenocarcinoma Based on TILs Grading

**DOI:** 10.3390/curroncol31120552

**Published:** 2024-11-25

**Authors:** Nur Rahmah Rasyid, Upik Anderiani Miskad, Muhammad Husni Cangara, Syarifuddin Wahid, Djumadi Achmad, Suryani Tawali, Mardiati Mardiati

**Affiliations:** 1 Department of Anatomical Pathology, Faculty of Medicine, Hasanuddin University, Makassar 90245, Indonesia; nurrahmahrasyid@unhas.ac.id (N.R.R.);; 2 Anatomical Pathology Laboratory, Hasanuddin University Hospital, Makassar 90245, Indonesia; 3 Department of Public Health, Faculty of Medicine, Hasanuddin University, Makassar 90245, Indonesia; suryani_tawali@med.unhas.ac.id

**Keywords:** colorectal cancer, tumor infiltrating lymphocytes, PD-1, PD-L1, immunotherapy, biomarkers

## Abstract

Aim: Colorectal cancer (CRC) is a prevalent malignancy with a high mortality rate. Tumor-infiltrating lymphocytes (TILs) play a crucial role in the immune response against tumors. Programmed death-1 (PD-1) and programmed death-ligand 1 (PD-L1) are key immune checkpoints regulating T cells in the tumor microenvironment. This study aimed to assess the relationships among PD-1 expression on TILs, PD-L1 expression in tumors, and TIL grading in colorectal adenocarcinoma. Methods: A cross-sectional design was employed to analyze 130 colorectal adenocarcinoma samples. The expression of PD-1 and PD-L1 was assessed through immunohistochemistry. A semi-quantitative scoring system was applied. Statistical analysis with the chi-square test was performed to explore correlations, with the data analyzed in SPSS version 27. Results: PD-1 expression on TILs significantly correlated with a higher TIL grading (*p* < 0.001), while PD-L1 expression in tumors showed an inverse correlation with TIL grading (*p* < 0.001). Conclusions: The expression of PD-1 on TILs and PD-L1 on tumor cells correlated significantly with the grading of TILs in colorectal adenocarcinoma. This finding shows potential as a predictive biomarker for PD-1/PD-L1 blockade therapy. Further studies are needed to strengthen these results.

## 1. Introduction

Colorectal cancer (CRC) is a widely occurring cancer globally, with high rates of incidence and mortality across numerous nations. [1,2,3]. This cancer originates from the mucosal lining of the colon and commonly progresses from normal mucosa to adenoma and carcinoma [4,5,6].

According to GLOBOCAN 2022, CRC is the third most prevalent cancer and the second highest cause of cancer-related mortality worldwide, following lung and breast cancers [1,2]. In Indonesia, CRC prevalence has also shown an increasing trend, partly attributed to lifestyle and dietary changes [1,2,7]. CRC currently ranks as the second most prevalent cancer among men, after lung cancer, and fourth among women, after breast, cervical, and ovarian cancers [1,2,8]. A local report from DR. Wahidin Sudirohusodo Hospital in Makassar Indonesia indicated that more than 150 colorectal adenocarcinoma cases were diagnosed between 2021 and 2023.

Globally, the number of new CRC cases is projected to rise to 3.2 million by 2040 due to aging populations and increasing life expectancy [1,2,3]. While most cases occur in older individuals, recent data show that CRC incidence is rising among younger populations as well [1,2,8]. Several studies have also observed a shift in the anatomical distribution of CRC incidence from the distal left colon to the proximal right colon, possibly due to more effective screening on the left side [4,8,9]. These trends call for enhanced strategies in prevention, screening, and treatment to reduce the overall burden of CRC [1,2,8].

Histologically, adenocarcinoma is the most common CRC subtype, accounting for more than 90% of cases [4,5,10]. Within the tumor microenvironment, tumor-infiltrating lymphocytes (TILs) are essential for the immune response against tumors, especially in inhibiting tumor progression [11,12,13]. CD8+ cytotoxic T cells, a major component of TILs, are crucial in recognizing and destroying tumor cells that express tumor antigens on MHC class I molecules [13,14]. The grading of TILs, based on their presence in the tumor stroma, has emerged as a valuable tool in assessing the immune landscape of CRC and its response to treatment [15,16].

Several studies have shown that the increased infiltration of TILs is associated with a better prognosis, as CD8+ T cells and NK cells play a role in limiting tumor growth or preventing metastasis [11,13,15]. However, many tumors develop mechanisms to evade the host immune response [9,17]. Programmed death-1 (PD-1) and its ligand, programmed death-ligand 1 (PD-L1), play a key role in this process [17,18,19].

PD-1 is a regulatory molecule that is expressed on the surface of activated T cells, B cells, and NK cells [18,19,20]. PD-1 expression on T cells can be upregulated in response to persistent antigen exposure, becoming a marker of T-cell exhaustion [19,20,21]. Its ligand, PD-L1, is expressed on tumor cells, T cells, B cells, and macrophages [11,18]. The interaction between PD-L1 and PD-1 delivers inhibitory signals that reduce cytokine production and T-cell proliferation, ultimately triggering T-cell apoptosis [19,20,22].

PD-L1 expression in tumors has been identified as a predictive biomarker for the response to PD-1/PD-L1-based immunotherapy in various cancers [23,24,25]. Despite these findings, the relationship between PD-1/PD-L1 expression and TILs in colorectal adenocarcinoma remains unclear. Does the expression of PD-1 and PD-L1 correlate with TIL grading in CRC? Can these markers serve as reliable indicators for predicting prognosis and response to immunotherapy?

This study aimed to explore the correlation between the expression of PD-1 on TILs and PD-L1 on tumor cells and the TILs grading in colorectal adenocarcinoma. We hypothesize that high PD-1 expression on TILs and high PD-L1 expression on tumor cells are positively correlated with a higher TIL grading, which may be indicative of a worse prognosis in colorectal adenocarcinoma. Through this study [11,13,15], we hope to enrich the understanding of CRC immunology and contribute to the development of more effective and personalized therapeutic strategies, especially for the population in Indonesia and beyond.

## 2. Materials and Methods

### 2.1. Patients and Tissue Specimens

This study is a quantitative descriptive observational analytic study with a cross-sectional approach that aimed to explore the correlation between PD-1 expression on tumor-infiltrating lymphocytes (TILs) and PD-L1 expression on tumor cells with TILs grading in colorectal adenocarcinoma. The study was conducted at the Anatomical Pathology Laboratory, Faculty of Medicine, Hasanuddin University, Makassar, Indonesia, from January 2021 to December 2023. The study population comprised colorectal tumor tissue samples diagnosed as adenocarcinoma. Formalin-fixed, paraffin-embedded (FFPE) tissue blocks from surgically excised colon and rectal tumors were obtained and sent to the Anatomical Pathology Laboratory of Dr. Wahidin Sudirohusodo Hospital and the Pathology Diagnostic Centre in Makassar. Samples were selected through purposive sampling to meet the inclusion criteria: confirmed adenocarcinoma diagnosis based on Hematoxylin–Eosin (HE) staining by two independent pathologists. Biopsy-derived samples or insufficient tissue for further analysis were excluded.

### 2.2. Assessment of Tumor-Infiltrating Lymphocytes (TILs)

TILs were assessed based on their presence and distribution in the tumor stroma using a semi-quantitative method in accordance with the recommendations of the International TILs Working Group [15,26]. TILs were measured on an ordinal scale based on the number of infiltrating lymphocytes present in the tumor tissue. TILs were graded on an ordinal scale as low (<10% of tumor stroma occupied by lymphocytes), medium (10–40%), or high (>40%) based on the proportion of lymphocytes within the tumor stroma. Assessment was conducted across five high-power fields (HPF) by two pathologists.

### 2.3. Immunohistochemical (IHC) Staining

To detect PD-1 and PD-L1 expression in tumor tissues, immunohistochemical (IHC) staining was performed using rabbit monoclonal antibodies, anti-PD-1 (EP239) and anti-PD-L1 (28-8), both obtained from Abcam (Cambridge, UK). These antibodies were validated for protein detection in FFPE tissues. Tissue sections (3–4 µm) were deparaffinized in xylene and rehydrated through a graded alcohol series. Epitope retrieval was performed by heating sections at 95 °C in a high-pH (Tris-EDTA, pH 9.0) buffer solution for 20 min. After cooling, the sections were incubated with the primary antibodies for 30 min at room temperature, and DAB (3,3′-diaminobenzidine) chromogen was applied to visualize antigen-antibody complexes. The sections were counterstained with hematoxylin to visualize tissue architecture. The stained slides were examined under an Olympus CX-43 light microscope by two pathologists. Antibody concentrations were applied according to the manufacturer’s protocol (Abcam, Cambridge, UK). This method allowed for a semi-quantitative analysis of PD-1 and PD-L1 expression in the tumor tissues.

### 2.4. PD-1 and PD-L1 Expression Assessment

- PD-1 expression was analyzed using a semi-quantitative immunoreactive score (IRS) system, calculated by summing the proportion score (PS) and intensity score (IS). The PS was determined based on the percentage of positively stained lymphocytes: 0 (<5%), 1 (5–10%), 2 (11–25%), 3 (26–50%), and 4 (>50%). The IS was based on the intensity of lymphocyte membrane staining: 0 (no staining), 1 (weak staining), 2 (moderate staining), and 3 (strong staining). The total IRS ranged from 0 to 7, with strong PD-1 expression defined as IRS ≥ 3 [27]. To ensure consistency, the scores were confirmed by two independent pathologists.
- PD-L1 expression was also assessed using the IRS system. The PS was calculated based on the percentage of tumor cells with positive staining: 0 (none), 1 (<1%), 2 (1–10%), 3 (11–50%), and 4 (>50%). The IS for PD-L1 was based on the intensity of membrane staining on tumor cells: 0 (no staining), 1 (weak staining), 2 (moderate staining), and 3 (strong staining). The total IRS ranged from 0 to 7, with strong PD-L1 expression defined as IRS ≥ 3 [16]. The scoring process was similarly verified by two independent pathologists to minimize inter-observer variability.

### 2.5. Statistical Analysis

Data were analyzed using SPSS version 27.0 software for Windows. Univariate analysis was performed to describe the demographic and clinical characteristics of the samples. Bivariate analysis using the Chi-Square test was used to evaluate the association between PD-1 and PD-L1 expression and TILs grading. The significance threshold used was *p*-value ≤ 0.05.

### 2.6. Data Availability and Ethics Approval

The data of this study will be made available to readers upon request, and all data supporting the findings in this study are stored in a public repository in accordance with MDPI policy. This study was approved by the Research Ethics Committee of the Faculty of Medicine, Hasanuddin University, with the following ethics approval code number: 434/UN4.6.4.5.31-/PP36/2A24 which was granted prior to the commencement of the study.

## 3. Results

### 3.1. Sample Characteristics

This study analyzed a total of 130 colorectal adenocarcinoma samples.

Table 1 shows the basic demographic and clinical characteristics of the patients included in this study. Most of the patients (63.8%) were older than 50 years, with a male-to-female ratio of approximately 1.13:1 (53.1% male and 46.9% female). Regarding the histopathological assessment, most of the samples (75.6%) were classified as low-grade adenocarcinoma, while 24.4% were classified as high-grade. In terms of tumor-infiltrating lymphocytes (TILs), 33.1% of the samples had a low TILs grading, 51.5% had an intermediate grading, and 15.4% had a high TILs grading. Lymphovascular invasion was observed in 16.2% of the samples, while 26.9% of the cases had lymph node metastasis. In terms of tumor budding, 43.1% of the samples had a high-grade tumor budding, followed by 40.8% with an intermediate-grade budding, and 16.2% with a low-grade budding. The classification of tumor invasion (pT) showed that most of the tumors (66.2%) were classified as pT2 (tumor invading the muscularis propria), followed by 32.3% classified as pT3 (tumor invading the subserosa), and 1.5% classified as pT4 (tumor invading other organs or structures). The tumor location was predominantly in the left (distal) colon (47.7%), followed by the right (proximal) colon (33.1%), the rectum (16.2%), and the rectosigmoid (3.1%).

### 3.2. TILs Assessment in 130 CRCs

**Figure 1:**
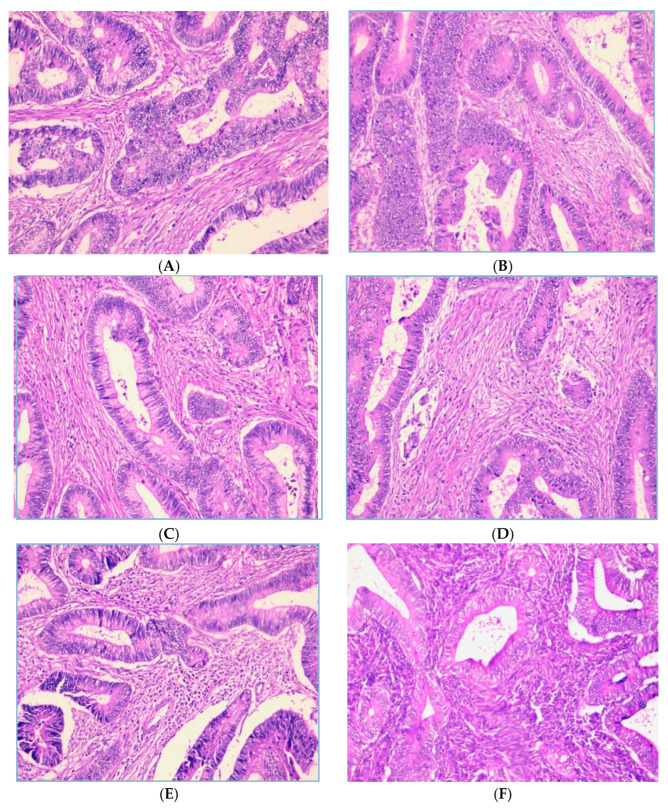
Representative images of TILs score (H.E, 200× magnification). Low-grade TILs with scores of 5% (**A**) and 10% (**B**)**.** Intermediate-grade TILs with scores of 20% (**C**) and 40% (**D**)**.** High-grade TILs with scores of 80% (**E**) and 90% (**F**).

The distribution of the tumor-infiltrating lymphocytes (TILs) grade shows that out of 130 samples, most had a moderate TILs grade. A total of 67 samples (51.5%) belonged to TILs grade 2 (moderate), while 43 samples (33.1%) belonged to TILs grade 1 (low). Only 20 samples (15.4%) were in TILs grade 3 (high). These data indicate that most patients had TILs infiltration at a moderate level. The representative figures of TILs shown in Figure 1.

### 3.3. Expression of PD-1 on TILs and PD-L1 on Tumor in 130 CRCs

**Figure 2:**
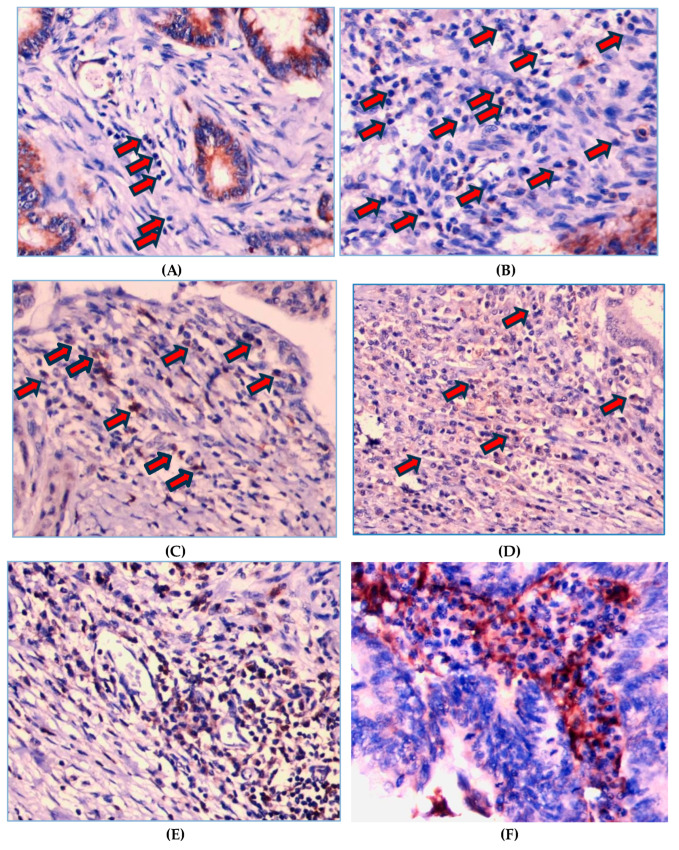
Representative images of immunohistochemistry intensity staining for PD-1 on TILs. Score 0 means not stained on lymphocyte cells (**A**) and score 1 is faintly stained on the membrane and/or cytoplasm of lymphocyte cells (**B**). Score 2 means PD-1 is stained on the membrane and/or cytoplasm of lymphocyte cells with moderate intensity, i.e., yellowish color (**C**,**D**). Score 3 means PD-1 is stained wholly or partially circularly on the membrane surface and/or cytoplasm of lymphocyte cells with high intensity, i.e., a light brown color (**E**,**F)**. The arrows indicate the intensity of PD-1 staining.

**Figure 3:**
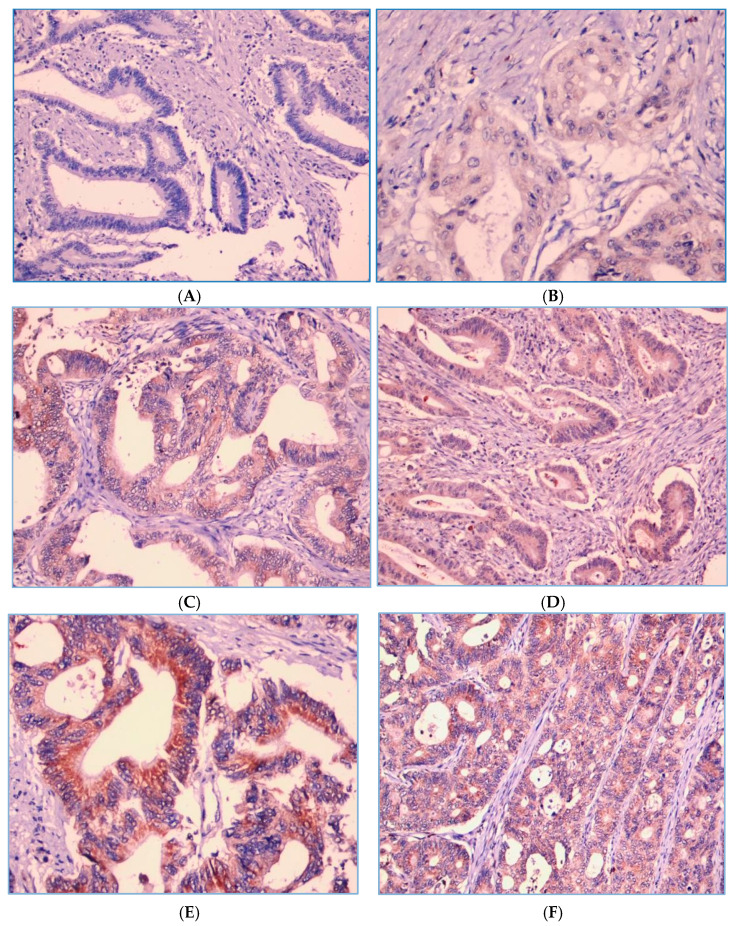
PD-L1 expression intensity score in the tumor cells. Score 0 means not stained in tumor cells (**A**) and score 1 means faintly stained in the membrane and/or cytoplasm of tumor cells (**B**). Score 2 means PD-L1 is stained in the membrane and/or cytoplasm of tumor cells with moderate intensity, i.e., yellowish color (**C**,**D)**. Score 3 means PD-L1 is stained wholly or partially circularly on the membrane and/or cytoplasm of tumor cells with high intensity, i.e., a light brown color (**E**,**F**).

As shown in Figure 2, PD-1 expression on TILs is localized on the cell membrane and/or the cytoplasm of lymphocyte cells. Of the 130 patients, there were 109 (83.8%) cases with a strong PD-1 expression and 21 (16.2%) cases with a weak PD-1 expression. Figure 3 shows that PD-L1 expression on tumor cells was also assessed at the cell membrane and/or the cytoplasm of tumor cells, with 115 (88.5%) cases showing a strong PD-L1 expression and 15 cases (11.5%) with a weak tumor PD-L1 expression.

### 3.4. Correlation of PD-1 Expression of TILs with Grading of TILs

Table 2 demonstrates the relationship between the total PD-1 immunostaining score of tumor-infiltrating lymphocytes (TILs) and the grading of TILs infiltration. A significant positive correlation was observed between the total PD-1 immunostaining score and TILs grading (*p* = 0.008).

Out of the total 130 colorectal adenocarcinoma samples, 109 samples (83.8%) exhibited a strong PD-1 expression on TILs, with most of these samples (55.1%) being classified as having an intermediate TILs grading, followed by 27.5% with a low grading and 17.4% with a high grading. On the other hand, samples with a weak PD-1 expression on TILs were predominantly associated with a low TILs grading (61.9%), while 33.3% showed an intermediate grading, and only 4.8% had a high grading. These data suggest that a higher PD-1 expression is more frequently associated with an increased TILs infiltration, indicating an active immune response. However, when PD-1 expression is weak, most samples tend to show a lower TILs infiltration. Then, the two tables below are the analysis table of the PD-1 expression proportion score and the analysis table of the PD-1 intensity score with TILs grading.

Table 3 presents the relationship between the PD-1 expression proportion score on tumor-infiltrating lymphocytes (TILs) and TILs grading. A statistically significant association was found between the PD-1 expression proportion score and the grading of TILs (*p* < 0.001). Of the 130 samples analyzed, those with no PD-1 expression (proportion score 0.00) predominantly displayed a low TILs grading, with 66.7% of these samples falling into the low-grade category. Conversely, as the PD-1 proportion score increased, there was a trend toward a higher TILs grading. For example, 45.7% of the samples with a PD-1 proportion score of 3.00 exhibited an intermediate TILs grading, while 28.6% showed a high-grade TILs infiltration. Notably, all samples with the highest PD-1 proportion score (4.00) were associated with a high TILs grading, with 100% of these samples showing high infiltration. In contrast, a lower PD-1 expression was more prevalent in samples with a lower TILs grading. These results suggest that an increase in PD-1 expression proportion is correlated with a higher grading of TILs infiltration. This relationship highlights the potential role of PD-1 in modulating TILs activity and the tumor’s immune microenvironment, where a higher proportion of PD-1 expression corresponds to a more pronounced immune infiltration.

Table 4 shows the relationship between the PD-1 expression intensity score on tumor-infiltrating lymphocytes (TILs) and TILs grading. Although there is a trend toward a higher TILs grading with a stronger PD-1 expression intensity, the relationship did not reach statistical significance (*p* = 0.109). Among the samples analyzed, 69.2% of those with an unstained PD-1 expression intensity (score 0) had a low TILs grading, while 30.8% displayed an intermediate TILs grading. A weak PD-1 expression intensity was most associated with an intermediate TILs grading (54.5%) and a low grading (36.4%). As the intensity score increased, more samples showed intermediate and high TILs gradings. For instance, 55.2% of the samples with a moderate PD-1 expression intensity (score 2) had a intermediate TILs grading, while 13.8% had a high grading. Among samples with a strong PD-1 expression intensity (score 3), 53.2% had an intermediate grading, and 19.5% exhibited a high grading. Despite the trend observed, the *p*-value of 0.109 indicates that there is no statistically significant correlation between the PD-1 intensity score on TILs and the grading of TILs infiltration in this study.

### 3.5. Correlation of Tumor PD-L1 Expression with Grading of TILs

Table 5 presents the relationship between the total PD-L1 immunostaining score in tumor cells and the grading of tumor-infiltrating lymphocytes (TILs). A strong PD-L1 expression in tumor cells was observed in 115 samples (88.5%), with most of these cases found in samples with low and intermediate TILs gradings (42 samples, 36.5%, and 64 samples, 55.7%, respectively). Only nine samples (7.8%) with a high TILs grading exhibited a strong PD-L1 tumor expression. Conversely, a weak PD-L1 tumor expression was predominantly observed in samples with a high TILs grading, accounting for 11 samples (73.3%). A weak expression was less common in samples with a low or intermediate TILs grading (one sample, 6.7%, and three samples, 20%, respectively). The statistical analysis using the Chi-Square test revealed a highly significant inverse relationship between PD-L1 tumor expression and TILs grading, with a *p*-value of less than 0.001. This suggests that as PD-L1 expression in a tumor increases, the grading of infiltrating TILs tends to decrease, and vice versa. Then, the two tables below are the analysis table of the PD-L1 expression proportion score and the analysis table of the PD-L1 intensity score with TILs grading.

Table 6 highlights the relationship between the proportion score of PD-L1 expression in tumor cells and the grading of tumor-infiltrating lymphocytes (TILs). Tumor samples with a PD-L1 proportion score of 4 (indicating high PD-L1 expression) were found predominantly in samples with low and intermediate TILs gradings (19 samples, 39.6%, and 26 samples, 54.2%, respectively). Only three samples (6.3%) with a high TILs grading exhibited a high PD-L1 proportion score. On the other hand, samples with a PD-L1 proportion score of 0 (indicating no PD-L1 expression) were mostly found in samples with a high TILs grading (10 samples, 71.4%). Only one sample (7.1%) with a low TILs grading and three samples (21.4%) with an intermediate TILs grading had no PD-L1 expression. The statistical analysis using the Chi-Square test showed a highly significant inverse relationship between the PD-L1 proportion score in tumor cells and the grading of TILs, with a *p*-value of less than 0.001. This indicates that a lower PD-L1 expression in tumor cells is associated with a higher TILs grading, and vice versa.

Table 7 presents the relationship between the intensity score of PD-L1 expression in tumor cells and the grading of tumor-infiltrating lymphocytes (TILs). Tumor samples with an unstained PD-L1 expression (score 0) were mostly found in samples with a high TILs grading (10 samples, 71.4%), with fewer occurrences in the samples with intermediate (three samples, 21.4%) and low TILs gradings (one sample, 7.1%). On the other hand, samples with strong PD-L1 intensity scores were predominantly found in samples with a low (18 samples, 35.3%) and intermediate (27 samples, 52.9%) TILs grading. Only six samples (11.8%) with a high TILs grading exhibited a strong PD-L1 expression. The statistical analysis using the Chi-Square test demonstrated a significant negative relationship between the intensity score of PD-L1 expression in tumor cells and the TILs grading, with a *p*-value of 0.001. This result suggests that a higher PD-L1 intensity expression in tumor cells is associated with a lower TILs grading, and vice versa.

### 3.6. The Correlation of PD-1 TILs and PD-L1 Tumor Expression with TILs Grade and Clinicopathological Parameters in 130 CRCs

As shown in Table 8, the expression of PD-1 on TILs and PD-L1 on tumor cells showed PD-1 expression (*p* = 0.008) and tumor PD-L1 expression (*p* < 0.001), which proved a significant correlation with the TILs grading, where a strong PD-1 expression was more common in samples with a high TILs infiltration rate and a weak PD-L1 expression was more common in samples with a high TILs grading. However, no significant correlation was found based on age, gender, histology grade, histology type, metastasis to lymph nodes, lymphovascular invasion, tumor budding grade, or depth of tumor invasion (*p* > 0.05).

## 4. Discussion

This study shows that the expression of PD-1 on TILs and PD-L1 on tumor cells plays an important role in the regulation of immune responses in colorectal adenocarcinoma. From Table 2 and Table 3, through Chi Square correlation test with *p* value < 0.001 and *p* = 0.008, respectively, there is a statistically significant relationship between the proportion expression and total score of PD-1 immunostaining on TILs, which is directly proportional to the grading of TILs infiltration, where, when the PD-1 expression on TILs is low, the grading of TILs infiltration also tends to be low, and when PD-1 on TILs is high, the grading of TILs will also be high. This indicates that in proportion, PD-1 expression reflects the level of activation and functionality of TILs in the tumor environment [28,29,30]. PD-1 expression is induced after T-cell activation, so the presence of PD-1 indicates that the T cells have been activated and may have started migrating to the tumor site [9,19].

Through Table 3, there is a tendency that when the proportion of PD-1 expression on TILs is low, the infiltration grading of TILs also tends to be lower. This indicates that TILs that express low PD-1 are less able to effectively infiltrate the tumor, which could be due to not being fully activated or due to other factors that limit their migration into the tumor. This is in accordance with the literature showing that PD-1 expression on TILs is not only related to the regulation of T-cell activation but also to the ability of the cells to infiltrate the tumor; these cells migrate and infiltrate the tumor microenvironment [21,28,30]. Studies by Pauken and Wherry showed that T cells that do not express PD-1 at adequate levels exhibit suboptimal activation status, which reduces their effectiveness in migrating into tumors and performing their effector functions [19,31].

In addition, other research suggests that the activation status of T cells, indicated by PD-1 expression, plays an important role in the ability of T cells to infiltrate tumors [18,20,32]. Under-activated TILs may be caused by encountering barriers from the tumor microenvironment that hinder their effective infiltration [20,33]. Factors such as the presence of immunosuppressive cytokines, nutrient deprivation, and hypoxic conditions within the tumor microenvironment may contribute to the decreased infiltration ability of TILs [33,34,35].

Thus, these results are consistent with previous findings showing that PD-1 expression on TILs is an important indicator in the regulation of T-cell migration into tumors as well as in the mediation of effective immune responses against tumor cells [18,20,32]. A low PD-1 expression may reflect a lack of T-cell activation, resulting in decreased ability to infiltrate into the tumor microenvironment and decreased effectiveness of the antitumor immune response [18,21,30].

Furthermore, we found something different in Table 4, where, when the PD-1 intensity expression on TILs increased, there was a tendency to increase the proportion of cases with a higher TILs grading score, but this difference did not reach statistical significance with *p* = 0.109. Thus, from Table 4, we cannot provide sufficient evidence to conclude a significant association between PD-1 intensity expression on TILs and TILs infiltration grading in colorectal adenocarcinoma. This could be due to several reasons rooted in the complexity of tumor biology and immune response in the tumor microenvironment [4,5]. These include variations in tumor heterogeneity and the tumor microenvironment at both the cellular and molecular levels and the presence of T-cell exhaustion [19,21,36]. This heterogeneity includes variations in gene expression, protein and cellular composition, and the response to antitumor therapy [9,37]. At the cellular level, this heterogeneity may include differences in proliferation, migratory ability, and ability to evade immune surveillance [21,38,39]. At the molecular level, this can involve variations in the expression of immune checkpoint molecules such as PD-1 on TILs as well as its ligand, PD-L1, on tumor cells [39,40].

This variability can result in significant differences in the infiltration pattern of TILs in different areas of the tumor within the same individual or between different patients [37,40]. As a result, PD-1 expression and the infiltration rate of TILs can be highly variable, causing difficulty in detecting a consistent relationship between PD-1 expression and the grading of TILs. For example, one part of the tumor may show a high PD-1 expression and a strong infiltration of TILs, while another part of the same tumor may show low expression and little infiltration of TILs [37,39,41].

Moreover, measurements made on this limited sample of heterogeneous tumors may not be fully representative of the overall tumor microenvironment, which may lead to inconsistent or statistically insignificant results. This poses a challenge in studies looking for relationships between factors such as PD-1 expression and the grading of TILs, as small samples or single point measurements cannot capture the full complexity of heterogeneous tumors [37,41].

In addition, the tumor microenvironment itself is highly dynamic and influenced by various factors such as immunosuppressive cytokines, nutrients, hypoxia, and interactions with different types of immunosuppressive cells, including Tregs, MDSCs (Myeloid- Derived Suppressor Cells), and tumor-associated macrophages (TAMs) [33,34,42]. These factors can further influence PD-1 expression on TILs as well as the ability of TILs to infiltrate and invade tumors, adding a layer of complexity in analyzing the relationship between PD-1 and TILs grading [20,21,34].

PD-1 is a classic marker of T-cell exhaustion, which is a condition in which T cells experience decreased function following persistent antigenic stimulation within the tumor microenvironment [19,20,32]. This T-cell exhaustion often occurs in tumors with a highly immunosuppressive environment, where T cells are constantly exposed to tumor antigens without being able to fully eliminate the tumor [19,32,34]. Thus, although there is a greater infiltration of T cells indicating the presence of mechanisms for T-cell activation and migration to the tumor site, this exhaustion leads to a decrease in the effector capabilities of T cells, including their ability to kill tumor cells and produce the pro-inflammatory cytokines required for an effective antitumor response [19,20,21].

In this context, a high-intensity PD-1 expression on TILs does not necessarily correlate with good effector function, even if the TILs have successfully infiltrated the tumor. Instead, T-cell exhaustion characterized by a high PD-1 expression can lead to T-cell dysfunction, where these cells are no longer able to optimally perform their tasks, such as tumor cell lysis or the secretion of cytokines required to maintain a sustained immune response [19,21,32]. Consequently, although grading TILs may indicate the presence of higher infiltration, the decreased effector function of T cells, resulting in an association between PD-1 intensity expression and TILs grading that does not show strong statistical significance [19,21].

Furthermore, T-cell exhaustion can affect various other aspects of immune function. For example, exhausted T cells tend to have decreased proliferation ability, cytokine secretion, and durability, all of which are important for effective antitumor effectors. These decreased capabilities may make exhausted TILs less effective in suppressing tumor growth, even though they may be present in large numbers [19,32]. Therefore, when viewed from the aspect of the PD1 expression intensity of TILs, a higher grading of TILs does not necessarily translate into a more effective immune response if the infiltrated T cells are in a state of exhaustion and not functioning optimally [20,21,43].

Thus, although TILs may express PD-1 and show signs of infiltration into the tumor, the presence of immunosuppressive elements in the tumor microenvironment may decrease the effectiveness of this infiltration, which may explain why the relationship between PD-1 expression intensity and TILs infiltration effectiveness is not always significant or consistent [11,33,42].

We also assessed the intensity, proportion, and total immunostaining score of tumor PD-L1 expression against the grading of TILs which can be seen in Table 5, Table 6 and Table 7. To further contextualize our findings, it is essential to consider the methodological choices made in assessing PD-L1 expression. The selection of Tumor Proportion Score (TPS) as the method for PD-L1 evaluation in this study was driven by the specific focus on tumor-cell expression in colorectal cancer. This approach aligns with our primary objective of analyzing PD-L1 directly on tumor cells, allowing for a targeted examination of immune checkpoint expression in the tumor microenvironment [16,44,45]. While the Combined Positive Score (CPS) method, which includes PD-L1 expression on both tumor cells and infiltrating lymphocytes, offers a broader perspective, our study aimed to maintain a focused and simplified analysis on tumor-cell PD-L1 expression alone [16,44]. From this table, there is an inverse relationship between PD-L1 expression in tumor cells and the grading of TILs, where, when PD-L1 expression in tumors is low, the grading of infiltrating TILs tends to be higher. This could indicate that tumor cells with a low PD-L1 expression may be less able to suppress the immune response, allowing more TILs to infiltrate the tumor. This is consistent with the theory that PD-L1 is one of the main mechanisms used by tumor cells to evade immune surveillance through interaction with the PD-1 receptor on T cells [11,18,24].

A high expression of PD-L1 on tumor cells allows them to bind PD-1 on TILs, sending negative signals that inhibit T-cell effector activities, including their proliferation, cytokine secretion, and cytotoxic ability [18,19,36]. As a result, tumor cells that express high PD-L1 can create an immunosuppressive microenvironment that prevents the infiltration and optimal function of TILs. When PD-L1 expression in tumors is low, these barriers are reduced, allowing more TILs to infiltrate the tumor and, potentially, attack tumor cells more effectively [11,18,33].

In addition, the results of this study also support the idea that tumors with a low PD-L1 expression may be more immunogenic, which can occur through several other pathways such as the innate immunity activation such as STING (stimulator of interferon genes) that can promote a strong immune response against tumors by increasing the production of pro-inflammatory cytokines and triggering the activation of dendritic cells and other immune cells [31,33,46]. This innate immune activation can transform TMEs to be more pro-inflammatory, increase antigen cross-presentation, and induce effector T cells without relying on PD-1/PD-L1 signaling, thus triggering a stronger immune response. Even without a high PD-L1 expression, tumor cells are more susceptible to detection by the immune system and are more easily destroyed by TILs [31,47]. This means that although tumor cells may attempt other immune evasion mechanisms, the lack of PD-L1 expression specifically makes them more susceptible to infiltration and attack by TILs [13,31,48].

Moreover, these data are in line with the concept that a high presence of TILs is often associated with a better prognosis in many types of cancer, including colorectal cancer. The ability of TILs to infiltrate into the tumor suggests that the patient’s immune system is actively involved in fighting tumor growth [11,13,15]. As a result, low PD-L1 expression in tumors not only allows more TILs to infiltrate but also contributes to better clinical outcomes by allowing the immune system to effectively suppress tumor growth [11,16,48].

The positive correlation between PD-1 expression on TILs and TIL grading in our findings is related to increased immune infiltration in line with the adaptive immune resistance mechanism, where TILs with an elevated PD-1 expression reflect an active immune response to tumor antigens in the tumor microenvironment [11,14,46]. However, we acknowledge that this relationship may not always be linear or consistent across all tumor environments [12,32]. Eiva et al. highlighted that other immune checkpoint molecules, such as CD39 and CD137, can modulate TIL behavior and may affect TIL infiltration independently of PD-1 expression [14]. This suggests that although PD-1 is a useful marker for immune activation, additional factors contribute to the complex dynamics of TIL infiltration in colorectal cancer [12,19,32].

In addition, Ruan et al. suggested that the immune response in colorectal cancer may vary depending on the level of immune checkpoint activation and the immunosuppressive landscape of the tumor [20,49]. In some cases, a higher PD-1 expression in TILs does not necessarily correlate with the effective targeting of tumor cells due to fatigue conditions caused by chronic antigen exposure within the tumor microenvironment [19,20,21]. This highlights that although PD-1 expression often parallels TIL density, certain conditions within the tumor microenvironment can disrupt this relationship, which emphasizes the need for the comprehensive profiling of immune checkpoint expression to fully understand immune dynamics in colorectal adenocarcinoma [12,19,49].

These findings not only provide insight into the immune mechanisms underlying colorectal cancer, but also have significant implications for patient management and treatment personalization [48,50]. The expression of PD-1 on TILs and PD-L1 on tumor cells indicates that these markers may play an important role in the clinical management of colorectal cancer [24,48]. A high expression of PD-1 and PD-L1 not only reflects the activity of the immune system in response to the tumor, but also suggests that these immune checkpoint pathways may be effective therapeutic targets [11,48,51]. In addition, the study by Yang et al. identified PD-L1 as a prognostic marker in colorectal cancer, where high PD-L1 expression was associated with decreased overall survival and disease-free survival [8,16,52]. This confirms that PD-L1 is not only involved in the biological process of tumor progression but also has substantial predictive, prognostic value for patients and guide treatment strategies [48,50].

PD-1 expression on TILs and PD-L1 on tumors can be used as predictive biomarkers to assess response to PD-1/PD-L1 blockade-based immunotherapies, such as pembrolizumab or nivolumab [48,50,51]. The use of these biomarkers could potentially help identify patients who are more likely to respond to therapy and improve clinical outcomes in CRC patients [24,48,51]. In addition, by assessing TILs, clinicians can more accurately predict patient prognosis and design more personalized therapeutic strategies [11,12,53]. By knowing the expression levels of these markers, clinicians can better predict the likelihood of a positive response to immunotherapy and select candidates who are more likely to benefit [12,22,54]. Patients with a high PD-1 and PD-L1 expression may have a worse prognosis and may require a more aggressive therapeutic approach or more intensive monitoring and are better candidates for PD-1/PD-L1 blockade-based immunotherapy, which has been shown to be effective in enhancing the immune response against tumors in various cancers, including melanoma and lung cancer [22,23,54]. In the long term, this approach may improve clinical outcomes and reduce the use of less effective treatments in patients who may not respond to PD-1/PD-L1-based immunotherapy [22,55,56].

Although these findings provide a strong indication of the clinical potential of PD-1 and PD-L1, further studies are needed to validate their clinical relevance in a wider patient population. Meanwhile, this study has several limitations that need to be considered. First, the sample size used in this study is relatively limited, which may affect the statistical power of the analysis performed. Second, the cross-sectional nature of the study may not fully capture the temporal dynamics between PD-1/PD-L1 expression and tumor progression over time. In addition, this study only included samples from one center, so the results may not be fully generalizable to a wider population. To overcome these limitations, larger studies and further research with a prospective design and involving larger populations from various centers need to be conducted to confirm the role of PD-1 and PD-L1 as prognostic biomarkers that can be applied in clinical practice as a tool to guide optimal therapeutic options for colorectal cancer patients. Future research may also explore the effects of PD-1/PD-L1 blockade-based therapies in patients with high PD-1/PD-L1 expression, as well as incorporate the Combined Positive Score (CPS) method to assess PD-L1 expression more comprehensively by including both tumor cells and immune cells in the tumor microenvironment. Studies assessing changes in PD-1/PD-L1 expression after immunotherapy are also important to identify potential resistance mechanisms, improve our understanding of immune checkpoint pathways, and refine therapeutic strategies for colorectal cancer.

## 5. Conclusions

This study shows that PD-1 expression on tumor-infiltrating lymphocytes (TILs) has a significant positive correlation with the grading of TILs in colorectal adenocarcinoma. The higher the grading of TILs, the stronger the PD-1 expression, reflecting T-cell exhaustion due to interaction with the tumor. In contrast, PD-L1 expression on tumor cells showed a negative correlation with TILs grading, where the lower the TILs grading, the stronger the PD-L1 expression on the tumor. These findings highlight the important role of the PD-1/PD-L1 pathway in the tumor microenvironment, as well as its potential as a therapeutic target in the management of colorectal cancer.

A potential clinical application of this study is the use of PD-1 and PD-L1 as predictive biomarkers to identify patients who may respond to PD-1/PD-L1 blockade-based immune therapy. Further studies with larger populations and prospective designs are required to strengthen these findings, as well as to explore the effectiveness of therapies targeted at the PD-1/PD-L1 pathway.

## Figures and Tables

**Table 1:** Basic Characteristics of Respondents.

Characteristics	Sample Quantity (n = 130)	Percentage (%)
**Age**		
≤ 50 years	47	36.2
>50 years	83	63.8
**Gender**		
Male	69	53.1
Women	61	46.9
**Histopathological Assessment**		
Low	101	77.6
High	29	22.4
**TIL Assessment**		
Low	43	33.1
Intermediate	67	51.5
High	20	15.4
**Lymphovascular Invasion**		
No.	109	83.8
Yes.	21	16.2
**Lymph Node Metastasis**		
No.	95	73.1
Yes.	35	26.9
**Tumor Budding**		
Low	21	16.2
Intermediate	53	40.8
High	56	43.1
**Classification pT**		
pTis	0	0
pT1	0	0
pT2	86	66.2
pT3	42	32.3
pT4	2	1.5

**Table 2:** Relationship between total PD-1 immunostaining score of tumor-infiltrating lymphocytes (TILs) and grading of tumor-infiltrating lymphocytes (TILs).

Variable	TILs Assessment				Total N (%)	*p*
		Low N (%)	Intermediate N (%)	High N (%)		
TIL PD-1 Immunostaining Score	Strong	30 (27.5)	60 (55.1)	19 (17.4)	109 (100.0)	0.008 *
	Weak	13 (61.9)	7 (33.3)	1 (4.8)	21 (100.0)	

* Statistically significant if the *p*-value is less than 0.05.

**Table 3:** Relationship between PD-1 expression proportion score of tumor-infiltrating lymphocytes (TILs) and grading of tumor-infiltrating lymphocytes (TILs).

Variable	TILs Assessment				Total N (%)	*p*
		Low N (%)	Intermediate N (%)	High N (%)		
TIL Proportion Score PD-1	0.00	10 (66.7)	5 (33.3)	0 (0.0)	15 (100.0)	<0.001 *
	1.00	22 (35.5)	35 (56.5)	5 (8.1)	62 (100.0)	
	2.00	2 (15.4)	11 (84.6)	0 (0.0)	13 (100.0)	
	3.00	9 (25.7)	16 (45.7)	10 (28.6)	35 (100.0)	
	4.00	0 (0.0)	0 (0.0)	5 (100.0)	5 (100.0)	

* Statistically significant if the *p*-value is less than 0.05.

**Table 4:** Relationship between PD-1 expression intensity score of tumor-infiltrating lymphocytes (TILs) and grading of tumor-infiltrating lymphocytes (TILs).

Variable	TILs Assessment				Total N (%)	*p*
		Low N (%)	Intermediate N (%)	High N (%)		
TILs Intensity Score PD-1	Unstaining	9 (69.2)	4 (30.8)	0 (0.0)	13 (100.0)	0.109 *
	Weak	4 (36.4)	6 (54.5)	1 (9.1)	11 (100.0)	
	Moderate	9 (31.0)	16 (55.2)	4 (13.8)	29 (100.0)	
	Strong	21 (27.3)	41 (53.2)	15 (19.5)	77 (100.0)	

* Statistically significant if the *p*-value is less than 0.05.

**Table 5:** Relationship between total tumor PD-L1 immunostaining score and tumor-infiltrating lymphocytes (TILs) grading.

Variable	TILs Assessment				Total N (%)	*p*
		Low N (%)	Intermediate N (%)	High N (%)		
PD-L1 Tumor Immunostaining Score	Strong	42 (36.5)	64 (55.7)	9 (7.8)	115 (100.0)	<0.001 *
	Weak	1 (6.7)	3 (20)	11 (73.3)	15 (100.0)	

* Statistically significant if the *p*-value is less than 0.05.

**Table 6:** Relationship between tumor PD-L1 expression proportion score and grading of tumor-infiltrating lymphocytes (TILs).

Variable	TILs Assessment				Total N (%)	*p*
		Low N (%)	Intermediate N (%)	High N (%)		
Tumor PD-L1 Proportion Score	0.00	1 (7.1)	3 (21.4)	10 (71.4)	14 (100)	<0.001 *
	1.00	0 (0)	0 (0)	1 (100)	1 (100)	
	2.00	10 (52.6)	9 (47.4)	0 (0)	19 (100)	
	3.00	13 (27.1)	29 (60.4)	6 (12.5)	48 (100)	
	4.00	19 (39.6)	26 (54.2)	3 (6.3)	48 (100)	

* Statistically significant if the *p*-value is less than 0.05.

**Table 7:** Relationship between tumor PD-L1 expression intensity score and grading of tumor-infiltrating lymphocytes (TILs).

Variable	TILs Assessment				Total N (%)	*p*
		Low N (%)	Intermediate N (%)	High N (%)		
Tumor PD-L1 Intensity Score	Unstaining	1 (7.1)	3 (21.4)	10 (71.4)	14 (100.0)	<0.001 *
	Weak	10 (33.3)	17 (56.7)	3 (10)	30 (100.0)	
	Moderate	14 (40)	20 (57.1)	1 (2.9)	35 (100.0)	
	Strong	18 (35.3)	27 (52.9)	6 (11.8)	51 (100.0)	

* Statistically significant if the *p*-value is less than 0.05.

**Table 8:** Expression of PD-1 and PD-L1 and their correlation with clinicopathological parameters.

Variable	PD-1 TILs		*p*	PD-L1 Tumor		*p*	TILs			*p*	Total
	Strong	Weak		Strong	Weak		Low	Intermediate	High		
	N (%)	N (%)		N (%)	N (%)		N (%)	N (%)	N (%)		N (%)
**Age**											
≤50 y.o	42 (89.4)	5 (10.6)	0.299	43 (91.5)	4 (8.5)	0.598	18 (38.3)	26 (55.3)	3 (6.4)	0.096	47 (100)
>50 y.o	67 (80.7)	16 (19.3)		72 (86.7)	11 (13.3)		25 (30.1)	41 (49.4)	17 (20.5)		83 (100)
**Gender**											
Male	58 (84.1)	11 (15.9)	1.000	63 (91.3)	6 (8.7)	0.421	23 (33.3)	34 (49.3)	12 (17.4)	0.766	69 (100)
Female	51 (83.6)	10 (16.4)		52 (85.2)	9 (14.8)		20 (32.8)	33 (54.1)	8 (13.1)		61 (100)
**Histological Grade**											
Low	83 (82.2)	18 (17.8)	0.406	92 (91.1)	9 (8.9)	0.100	35 (34.7)	54 (53.5)	12 (11.9)	0.118	101 (100)
High	26 (89.7)	3 (10.3)		23 (79.3)	6 (20.7)		8 (27.6)	13 (44.8)	8 (27.6)		29 (100)
**Histological Type**											
Adenocarcinoma	102 (85.7)	17 (14.3)	0.253	104 (87.4)	15 (12.6)	0.236	38 (31.9)	61 (51.2)	20 (16.8)	0.396	119 (100)
Mucinous Adenocarcinoma	7 (63.6)	4 (36.4)		11 (100)	0 (0.0)		5 (45.5)	6 (54.5)	0 (0.0)		11 (100)
**Lymph Node Metastasis**											
Yes	28 (80)	7 (20)	0.649	30 (85.7)	5 (14.3)	0.547	11 (31.4)	16 (45.7)	8 (22.9)	0.351	35 (100)
No	81 (85.3)	14 (14.7)		85 (89.5)	10 (10.5)		32 (33.7)	51 (53.7)	12 (12.6)		95 (100)
**Lymphovascular Invasion**											
Yes	15 (71.4)	6 (28.6)	0.108	19 (90.5)	2 (9.5)	1.000	7 (33.3)	9 (42.9)	5 (23.8)	0.467	21 (100)
No	94 (86.2)	15 (13.8)		96 (88.1)	13 (11.9)		36 (33.0)	58 (53.2)	15 (13.8)		109 (100)
**Tumor Budding**											
Low	18 (85.7)	3 (14.3)	0.960	19 (90.5)	2 (9.5)	0.573	4 (19.0)	12 (57.1)	5 (23.8)	0.333	21 (100)
Intermediate	44 (83.0)	9 (17.0)		45 (84.9)	8 (15.1)		16 (30.2)	28 (52.8)	9 (17.0)		53 (100)
High	47 (83.9)	9 (16.1)		51 (91.1)	5 (8.9)		23 (41.1)	27 (48.2)	6 (10.7)		56 (100)
**pT**											
pT1	0 (0)	0 (0)	0.821	0 (0)	0 (0)	0.461	0 (0)	0 (0)	0 (0)	0.164	0 (0)
pT2	72 (83.7)	14 (16.3)		74 (86.0)	12 (14.0)		25 (29.1)	43 (50)	18 (20.9)		86 (100)
pT3	35 (83.3)	7 (16.7)		39 (92.9)	3 (7.1)		17 (40.5)	23 (54.8)	2 (4.8)		42 (100)
pT4	2 (100)	0 (0)		2 (100)	0 (0)		1 (50)	1 (50)	0 (0)		2 (100)
**TILs**											
Low	38 (37.3)	5 (17.9)	0.008 *	42 (36.5)	1 (6.7)	<0.001 *	/	/	/	/	43 (100)
Intermediate	53 (52.0)	14 (50.0)		64 (55.7)	3 (20.0)		/	/	/		67 (100)
High	11 (10.8)	9 (32.1)		9 (7.8)	11 (73.3)		/	/	/		20 (100)

* Statistically significant if the *p*-value is less than 0.05.

## Data Availability

The raw data used in this study will be made available by the authors upon request.
